# Predicting Intra- and Postpartum Hemorrhage through Artificial Intelligence

**DOI:** 10.3390/medicina60101604

**Published:** 2024-09-30

**Authors:** Carolina Susanu, Anamaria Hărăbor, Ingrid-Andrada Vasilache, Valeriu Harabor, Alina-Mihaela Călin

**Affiliations:** 1 Clinical and Surgical Department, Faculty of Medicine and Pharmacy, “Dunărea de Jos” University, 47 Domnească Street, 800008 Galati, Romania; carolina.susanu@ugal.ro (C.S.); valeriu.harabor@ugal.ro (V.H.);; 2 Department of Mother and Child Care, “Grigore T. Popa” University of Medicine and Pharmacy Iasi, 700115 Iasi, Romania

**Keywords:** postpartum hemorrhage, machine learning, algorithms

## Abstract

*Background and Objectives*: Intra/postpartum hemorrhage stands as a significant obstetric emergency, ranking among the top five leading causes of maternal mortality. The aim of this study was to assess the predictive performance of four machine learning algorithms for the prediction of postpartum and intrapartum hemorrhage. *Materials and Methods*: A prospective multicenter study was conducted, involving 203 patients with or without intra/postpartum hemorrhage within the initial 24 h postpartum. The participants were categorized into two groups: those with intra/postpartum hemorrhage (PPH) and those without PPH (control group). The PPH group was further stratified into four classes following the Advanced Trauma Life Support guidelines. Clinical data collected from these patients was included in four machine learning-based algorithms whose predictive performance was assessed. *Results*: The Naïve Bayes (NB) algorithm exhibited the highest accuracy in predicting PPH, boasting a sensitivity of 96.3% and an accuracy of 98.6%, with a false negative rate of 3.7%. Following closely were the Decision Tree (DT) and Random Forest (RF) algorithms, each achieving sensitivities exceeding 94% with a false negative rate of 5.9%. Regarding severity classification I, the NB and Support Vector Machine (SVM) algorithms demonstrated superior predictive capabilities, achieving a sensitivity of 96.4%, an accuracy of 92.1%, and a false negative rate of 3.6%. The most severe manifestations of HPP were most accurately predicted by the NB algorithm, with a sensitivity of 89.3%, an accuracy of 82.4%, and a false negative rate of 10.7%. *Conclusions*: The NB algorithm demonstrated the highest accuracy in predicting PPH. A notable discrepancy in algorithm performance was observed between mild and severe forms, with the NB and SVM algorithms displaying superior sensitivity and lower rates of false negatives, particularly for mild forms.

## 1. Introduction

Intra/postpartum hemorrhage represents a significant obstetric emergency [1]. Annually, approximately 500,000 maternal deaths occur due to pregnancy-related complications [2]. Intra/postpartum hemorrhage ranks among the top five causes of maternal mortality in both developed and low-resource settings, although the absolute risk of death from postpartum hemorrhage is substantially lower in high-income countries. This condition affects approximately 1–5% of births globally and remains the leading cause of maternal morbidity and mortality [2]. To address maternal hemorrhage effectively, management protocols have been developed, incorporating the prospective evaluation of postpartum hemorrhage risk [3]. These protocols aim to guide interventions in critical situations and offer prophylactic measures for high-risk patients.

Research indicates that postpartum blood loss is frequently underestimated in clinical settings, with the actual incidence being considerably higher than reported. It is estimated that 54–93% of postpartum hemorrhage cases could be prevented [4]. Due to the frequent inaccuracy of blood loss estimations, accurate prognosis and anticipation of postpartum hemorrhage remain challenging. Early recognition of this condition, immediate access to medical resources, and prompt clinical intervention are critical in preventing maternal mortality and severe morbidity.

In 2017, the American College of Obstetricians and Gynecologists (ACOG) updated the definition of postpartum hemorrhage (PPH) as follows [3]: cumulative blood loss ≥ 1000 mL, or bleeding accompanied by signs or symptoms of hypovolemia within 24 h of birth. This revised definition is applicable to both vaginal and cesarean deliveries. Nevertheless, ACOG highlighted that blood loss exceeding 500 mL during vaginal delivery should still be regarded as abnormal, particularly if heavy bleeding continues, warranting thorough evaluation and close monitoring by the medical team.

On the other hand, the newer definition of postpartum hemorrhage, as stated in the FIGO (International Federation of Gynecology and Obstetrics) guideline, is represented by an estimated blood loss of more than 500 mL within 24 h of a vaginal birth or 1000 mL after cesarean section, or any blood loss sufficient to compromise hemodynamic stability [4].

Despite the advancements in obstetric care, intra/postpartum hemorrhage (PPH) remains a critical concern worldwide, particularly in low-resource settings where access to timely medical interventions may be limited.

The complexity of PPH management is compounded by its unpredictable nature, as hemorrhages can occur even in women with no identifiable risk factors during pregnancy. Traditional risk assessment models, although helpful, are often limited by their reliance on a small number of variables and fail to capture the complex interplay of factors that contribute to PPH.

A cohort study involving over 154,000 births, which examined 666 cases of postpartum hemorrhage (PPH), identified the following significant risk factors [5]: incomplete delivery of the placenta, failure of labor to progress, placental accreta spectrum disorder (PAS), lacerations, vaginal birth assisted with forceps or vacuum, macrosomic fetuses, hypertensive disorders, medically-induced labor, and dystocic labor.

The etiology of PPH is often categorized under the framework of the “4 Ts”: Tone: uterine atony; Trauma: laceration, rupture; Tissue: retained tissue, blood clots, or PAS; Thrombin: coagulopathy [6].

Although key risk factors for postpartum hemorrhage have been identified, the condition may still occur in patients without these clinical risk factors [7]. Consequently, it is essential to assess the risk of postpartum hemorrhage both during pregnancy and upon admission, with continuous reevaluation as labor progresses [8]. Current studies suggest that the accuracy of predicting major obstetric hemorrhage ranges between 60 and 85% [7]. Therefore, there is a pressing need for more cost-effective predictive models to improve the identification of patients at risk for postpartum hemorrhage.

In recent years, machine learning (ML) has emerged as a powerful tool for improving clinical decision-making, particularly in the field of obstetrics. By analyzing large datasets with numerous predictors, ML algorithms can detect patterns and interactions between variables that are not immediately apparent in traditional statistical models. This has led to the development of ML-based predictive models that outperform conventional methods in both accuracy and sensitivity, offering a more personalized approach to PPH risk assessment [8].

Given the high incidence of PPH and its associated morbidity and mortality, there is a clear need for more effective predictive tools. Machine learning offers an innovative solution by enabling the development of algorithms that can integrate a wide range of clinical and demographic factors, thereby improving early detection and allowing for timely interventions. The use of ML in predicting obstetric complications has shown promising results, especially for preeclampsia and intrauterine growth restriction [9,10,11,12]. In this context, further exploration of machine learning algorithms could lead to substantial improvements in maternal health outcomes, particularly in high-risk populations [13].

This study aims to contribute to the growing body of research on machine learning applications in obstetrics by evaluating the predictive accuracy of four ML algorithms—Naive Bayes; Decision Tree; Random Forest; and Support Vector Machine—for PPH prediction using clinical risk factors. These algorithms could help clinicians identify patients at high risk of developing postpartum hemorrhage, thus enabling timely and effective interventions that could reduce the incidence of PPH and ultimately save maternal lives.

## 2. Materials and Methods

We conducted a multicenter prospective study involving patients who experienced intra/postpartum hemorrhage within the first 24 h after birth. The study was carried out at “Buna Vestire” Obstetrics and Gynecology Hospital in Galati and “Cuza Vodă” Obstetrics-Gynecology Clinical Hospital in Iasi, from October 2022 to September 2023.

The study was conducted in compliance with the Declaration of Helsinki, and ethical approval was granted by the institutional review boards of both participating hospitals. Moreover, this study is part of the doctoral research of Carolina Susanu and received the ethical approval from the Institutional Ethics Committee of “Dunarea de Jos” University of Medicine and Pharmacy from Galati, Romania (no. 740/07.12.2022). All patients provided informed consent before their inclusion in this study, and patient anonymity was preserved throughout the research process.

Inclusion criteria were singleton pregnancies reaching at least 28 weeks of gestation, maternal age of 18 years or older, and the presence of at least one risk factor for postpartum hemorrhage. Exclusion criteria included multiple pregnancies, chromosomal or structural fetal malformations, first and second trimester abortions, and lack of informed consent.

Medical data were collected from medical records, encompassing demographic information, personal and family medical history, obstetric history, and birth outcomes and complications.

**Figure 1:**
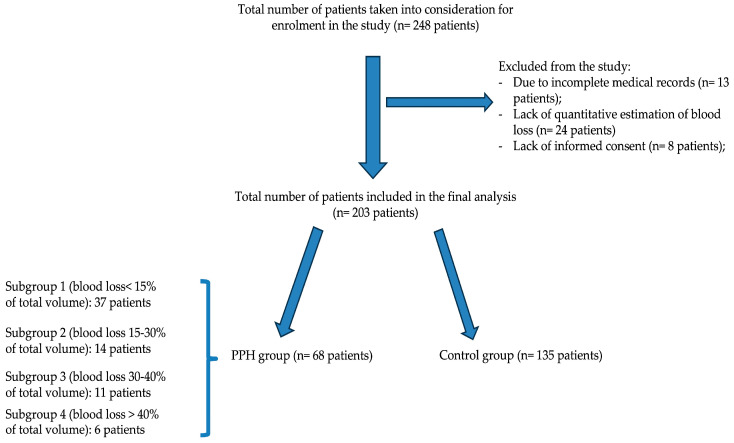
Flowchart of the study groups and subgroups.

The sample size and power analysis were performed before conducting this study using STATA SE software (version 18.5, released in 2024. StataCorp LLC, Lakeway Dr., College Station, TX, USA). The minimal sample size needed to reflect a 20% difference between PPH and control group was 143 patients, with a group allocation of 1:2 (exposed group—48 patients: control group—95 patients), an alpha error of 0.05, and a power of 80%. In the end, we enrolled 203 patients who met the inclusion criteria. A graphical representation of the patients’ segregation into the main study groups and subgroups is represented in Figure 1.

Initially, patients were categorized into two groups: those with postpartum hemorrhage (group 1, n = 68 patients) and those without postpartum hemorrhage (group 2, n = 135 patients), to identify differences between the groups using univariate analysis.

Subsequently, the first group was further classified into four subgroups based on the severity of postpartum hemorrhage (PPH) according to the Advanced Trauma Life Support (ATLS) manual [12]: subgroup 1: PPH < 15% (n = 37 patients), subgroup 2: PPH 15–30% (n = 14 patients), subgroup 3: PPH 30–40% (n = 11 patients), and subgroup 4: PPH > 40% (n = 6 patients). The blood loss estimation was quantitative using a volumetric container.

The clinical data from these subgroups, along with the control group, were utilized to develop four machine learning algorithms: decision tree (DT), naïve Bayes (NB), support vector machine (SVM), and random forest (RF). Sensitivity analysis was performed for each of these algorithms to evaluate their performance. The models were trained using 80% of the dataset, with the remaining 20% reserved for testing their predictive accuracy. Each algorithm was trained to predict both the occurrence of PPH and its severity based on input variables such as maternal history, obstetric factors, and labor characteristics.

The performance of each algorithm was assessed based on standard evaluation metrics, including accuracy, sensitivity, specificity, and the area under the curve (AUC) of the receiver operating characteristic (ROC). Additionally, the false positive and false negative rates were calculated to measure the reliability of the models in predicting PPH. A comparative analysis was performed between the machine learning algorithms and traditional statistical models to assess their relative performance in forecasting PPH.

For statistical analysis, chi-square tests were used to compare categorical variables between groups, while continuous variables were analyzed using independent t-tests. Logistic regression models were employed to identify independent risk factors for PPH. Statistical significance was set at *p* < 0.05, and all analyses were conducted using Python libraries (Version Python 3.12.1, Released 2023. Python Corp. Lacombe, LA, USA) and SPSS software (Version 29.0.2.0, Released 2023. IBM Corp., Armonk, NY, USA).

## 3. Results

Our study included a total of 203 patients, of whom 68 were diagnosed with postpartum hemorrhage (PPH), while the remaining 135 patients comprised the control group. The results of the univariate analysis are presented in Table 1.

The mean age and standard deviation of patients were 29.22 ± 6.88 years in group 1 and 28.62 ± 6.39 years in group 2. No significant statistical differences were observed regarding the patients’ living environments (*p* = 0.11). A higher incidence of nulliparous patients was encountered in the first study group (64.66%) compared to the control group (56.03%), but this difference did not reach statistical significance (*p* = 0.18).

Pre-existing maternal pathologies played a substantial role in the occurrence of obstetric hemorrhages. Among patients who experienced postpartum hemorrhage, 1.72% had a history of kidney disease, and 8.62% had a history of preeclampsia. Hypertension was noted in 8.62% of postpartum hemorrhage cases, compared to 0.86% in the control group. Additionally, 14.7% of mothers with macrosomic fetuses developed postpartum hemorrhage, in contrast to 2.2% in the control group. Obesity was prevalent in 26.72% of group 1, compared to 4.31% in the control group. Placental retention was diagnosed in 4.08% of postpartum hemorrhage cases versus 0.74% in the control group, and placental adhesion disorders were found in 10.2% of postpartum hemorrhage cases in group 1, compared to 0% in group 2.

**Figure 2:**
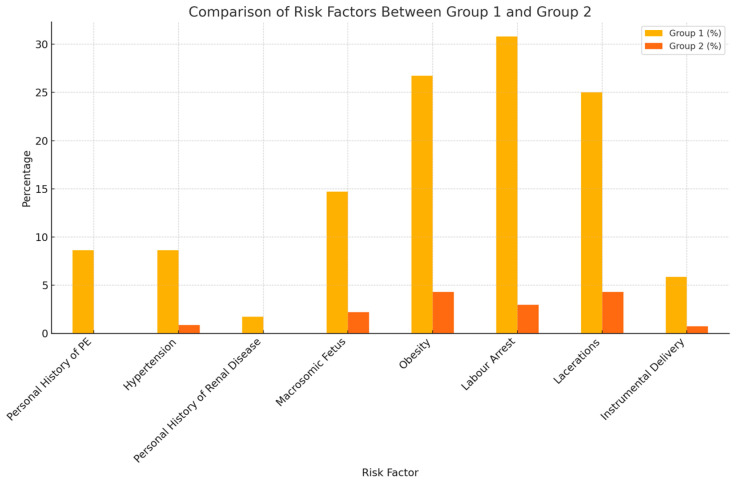
Comparison of risk factors between the main study groups.

In 30.8% of cases, labor failure was linked to postpartum hemorrhage. Vulvovaginal lacerations occurred in 25% of cases from the postpartum hemorrhage group, compared to 4.31% of cases included in the control group. Instrumental deliveries were used in 5.88% of postpartum hemorrhage cases and in 0.74% of cases from the control group. This data are outlined in Figure 2.

Our analysis revealed that patients who developed postpartum hemorrhage (PPH) had a significantly higher incidence of the following risk factors: personal history of preeclampsia and chronic hypertension (*p <* 0.001), obesity (*p <* 0.001), macrosomic fetuses (*p <* 0.001), placental retention (*p* = 0.001), placental accrete spectrum disorder (*p <* 0.001), instrumental delivery (*p =* 0.002), and vulvo-vaginal lacerations (*p <* 0.001).

Table 2 presents the results regarding the predictive performance of the four machine learning algorithms in predicting PPH and its severity. A total of 37 patients were included in the class 1 hemorrhage subgroup (54.4%), 14 patients were included in the class 2 hemorrhage subgroup (20.5%), 11 patients were included in the class 3 hemorrhage subgroup (16.1%), and 6 patients were included in the class 4 hemorrhage subgroup (8.8%) based on quantitative measurements.

**Figure 3:**
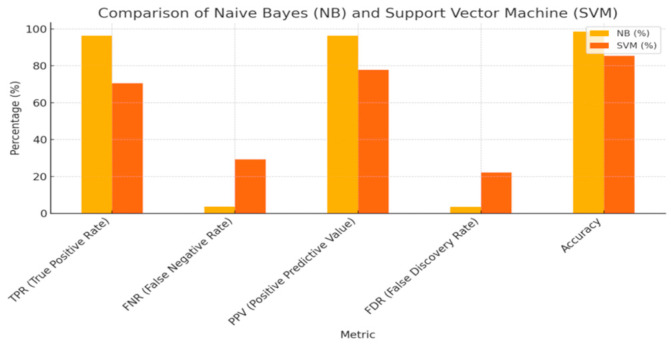
Comparison of Naive Bases (NB) and Support Vector Machine (SVM).

Our findings indicate that the Naïve Bayes (NB) algorithm most accurately predicted postpartum hemorrhage (PPH), achieving a sensitivity of 96.3% and an accuracy of 98.6%, with a false negative rate of 3.7%. The graphical representation of these predictive performances can be found in Figure 3.

This was closely followed by the Decision Tree (DT) and Random Forest (RF) algorithms, both demonstrating sensitivities above 94% and a false negative rate of 5.9%.

For the least severe forms of PPH, specifically severity class I, the NB and Support Vector Machine (SVM) algorithms exhibited the best predictive performance, with a sensitivity of 96.4%, an accuracy of 92.1%, and a false negative rate of 3.6%. Notably, all algorithms showed superior predictive capability for this less severe form of PPH compared to the more severe forms.

Conversely, the most severe forms of PPH were most accurately predicted by the NB algorithm, which achieved a sensitivity of 89.3%, an accuracy of 82.4%, and a false negative rate of 10.7%. The SVM and RF algorithms followed, with predictive accuracy ranging between 73.5% and 76.5%.

When compared to traditional logistic regression models, all four machine learning algorithms demonstrated superior accuracy and sensitivity. Logistic regression models achieved an overall accuracy of 85.2%, which was notably lower than that of the Naive Bayes and Random Forest models. Furthermore, the machine learning models were better at handling complex, nonlinear relationships between variables, which contributed to their higher predictive performance. The ability of these algorithms to integrate a larger number of predictors enhanced their capability to identify at-risk patients more accurately.

In a subgroup analysis, patients with risk factors such as hypertensive disorders, placental abnormalities, and macrosomic fetuses were particularly well predicted by the Naive Bayes and Random Forest models. These algorithms had sensitivity exceeding 95% in these high-risk subgroups. Patients with no identifiable risk factors posed a greater challenge for all models, although the Naive Bayes algorithm still managed to achieve an accuracy of 88.1% in predicting PPH for this group.

The results of this study demonstrate that the Naive Bayes algorithm consistently outperformed the other machine learning models across multiple performance metrics. It achieved the highest accuracy and sensitivity for predicting both the occurrence and severity of PPH, especially in high-risk subgroups.

The Decision Tree and Random Forest algorithms also showed strong performance, particularly in predicting the occurrence of PPH, though they were less accurate in classifying the severity of hemorrhage. The Support Vector Machine, while effective overall, showed lower predictive accuracy for severe PPH cases compared to the other models.

## 4. Discussion

The intricate process of predicting the risk of postpartum hemorrhage requires consideration of multiple factors, including the pregnant woman’s medical and personal history, the progression of pregnancy and labor, and the condition of the fetus. Given the substantial workload faced by clinicians, there is a risk that certain risk factors may be overlooked. The objective of this study was to support clinicians by utilizing machine learning algorithms to enhance risk prediction and significantly reduce the incidence of postpartum hemorrhage, thereby lowering maternal mortality rates.

In this study, we aimed to develop four machine learning algorithms for predicting postpartum hemorrhage (PPH) and its subtypes. Our results indicated that the Naïve Bayes (NB) algorithm provided the most accurate predictions for postpartum hemorrhage (PPH), followed closely by the Decision Tree (DT) and Random Forest (RF) algorithms. Notably, all algorithms demonstrated better performance in predicting PPH overall compared to its subtypes, suggesting a lower discriminatory ability for different postpartum hemorrhage (PPH) classes.

Furthermore, when assessing the algorithms’ performance in predicting specific postpartum hemorrhage (PPH) subtypes, we observed superior performance in predicting mild forms of PPH compared to severe forms, with the NB and Support Vector Machine (SVM) algorithms showing the highest sensitivity and lowest false negative rates.

Our findings align with recent studies. For instance, a prospective study evaluating the predictive performance of four machine learning algorithms using 55 risk factors for postpartum hemorrhage (PPH) found that the RF algorithm significantly outperformed logistic regression-based algorithms in terms of discriminatory and predictive ability [14]. A study conducted in the United States involving 200,000 cases utilized a logistic regression model to predict obstetric complications, demonstrating that timely and effective management of patients could be achieved based on these predictions [15]. In a comparative analysis between traditional statistical models and machine learning algorithms for predicting postpartum hemorrhage in a dataset of 8888 patients, artificial intelligence demonstrated significantly superior accuracy, sensitivity, and specificity [16].

A prospective study evaluated the predictive performance of algorithms based on logistic regression, Naive Bayes (NB), decision trees (DT), and random forests (RF) for the prediction of postpartum hemorrhage (PPH). The study found that the NB algorithm exhibited the best predictive performance, achieving an accuracy of 95%, a specificity of 97%, and an area under the curve (AUC) value of 0.76 [17]. Machine learning models can incorporate a wide range of algorithms, utilizing large datasets with complex features, enabling the combination of high accuracy, prediction quality, and sensitivity.

Liu et al., conducted a retrospective study that evaluated three machine learning models to predict postpartum hemorrhage after vaginal delivery in a cohort of 25,098 deliveries [18]. The models were represented by Random Forest, K Nearest Neighbor (KNN), and one model that was integrated with Lightgbm (LGB) and logistic regression (LR). These models were trained using 16 high-risk factors, and the best predictive performance was achieved by the LGB + LR model, with its sensitivity and specificity reaching 69% and 80%, respectively [18]. In contrast, the logistic regression model had an AUC value of 0.729, which was lower than other machine learning models. Moreover, the addition of a uterine contraction curve significantly increased the overall performance of all machine learning models, pointing out the need for the inclusion of paraclinical parameters in order to achieve better predictive performance.

In this study, we demonstrated that machine learning algorithms, particularly Naive Bayes, can significantly enhance the prediction of intra- and postpartum hemorrhage (PPH) compared to traditional statistical models. The superior performance of machine learning models, especially in high-risk groups such as patients with preeclampsia, macrosomic fetuses, and placental disorders, underscores their ability to capture complex, nonlinear interactions between multiple clinical variables.

The California Maternal Quality Care Collaborative (CMQCC) has developed an algorithm for postpartum hemorrhage (PPH) risk stratification based on maternal risk factors [19]. Patients are classified as low-risk if they have singleton pregnancies, no history of cesarean section or bleeding diathesis, and have had a maximum of four previous births. Risk factors that place patients in the medium-risk category include multiparity, prolonged labor, chorioamnionitis, or a history of hemorrhagic diathesis. The highest risk for postpartum hemorrhage (PPH) is associated with factors such as placental adhesion abnormalities, HELLP syndrome, fetal death in utero, or uterine rupture.

Machine learning offers significant advantages by enabling the development of automated models based on a large number of predictors and the ability to capture complex relationships between variables [20,21]. Its application has become increasingly widespread in the medical field, particularly for risk prediction. As such, predictive models generated through machine learning can assist clinicians in making informed decisions [14]. Recent studies have employed machine learning algorithms to predict postpartum hemorrhage, demonstrating the potential of these techniques [14,22]. Further research in this area could enhance clinical practice and contribute to the reduction of maternal mortality.

This highlights the limitations of conventional approaches that rely on a narrower set of risk factors. Our findings align with previous research showing that machine learning can outperform logistic regression in predicting obstetric complications, providing a more nuanced, individualized risk assessment.

One of the key strengths of this study lies in its multicenter, prospective design, which ensured a robust dataset reflective of real-world clinical conditions. The integration of machine learning into clinical practice could potentially reduce human error associated with high clinician workloads, offering a more reliable and consistent risk assessment for PPH. This, in turn, can enable early interventions, improve patient outcomes, and reduce the rate of maternal deaths associated with hemorrhagic complications.

However, several limitations must be acknowledged. The relatively small sample size and the limited number of risk factors considered may restrict the generalizability of the findings. Moreover, the low incidence of severe forms of PPH can constitute a bias of selection that reduces the overall accuracy of the evaluated models.

Future studies should aim to include larger and more diverse patient populations, incorporating additional clinical parameters to further refine the predictive accuracy of these models. Additionally, while machine learning offers great promise, its practical implementation in clinical settings requires careful consideration of infrastructure, training, and potential ethical concerns surrounding automated decision-making.

Despite their potential, implementing machine learning in clinical practice poses challenges, including the need for proper training, data integration, and addressing concerns over automated decision-making. Moreover, identifying the best clinical and paraclinical risk factors for disease predictions is a challenging issue that needs refining [23,24]. Future studies should focus on validating these algorithms across diverse populations and settings to ensure their reliability and generalizability.

## 5. Conclusions

This study demonstrates the potential of machine learning algorithms to significantly improve the prediction of intra- and postpartum hemorrhage.

The Naive Bayes algorithm was the most effective in predicting the evaluated outcomes, achieving the highest accuracy and sensitivity in predicting both the occurrence and severity of PPH. The algorithm’s ability to incorporate multiple clinical variables and detect complex interactions outperformed traditional logistic regression models.

Machine learning algorithms, particularly Naive Bayes, show considerable promise in improving the prediction and management of PPH. By enhancing the early detection of high-risk patients, these models have the potential to transform obstetric care, reduce maternal mortality, and contribute to safer childbirth outcomes.

Further research and validation in larger cohorts are essential to confirm these findings and facilitate the integration of machine learning into routine clinical practice for obstetric hemorrhage management.

## Figures and Tables

**Table 1:** Clinical characteristics of the main study groups.

Patient’s Characteristics	Group 1 (PPH, n = 68)	Group 2 (Without PPH, n = 135)	*p* Value
Age, years (mean ± SD)	29.22 ± 6.88	28.62 ± 6.39	0.49
Medium (n/%)	Urban = 32 (46.55%) Rural = 36 (53.45%)	Urban = 62 (45.69%) Rural = 73 (54.31%)	0.11
Parity (n/%)	Nulliparity = 44 (64.66%) Multiparity = 24 (35.34%)	Nulliparity = 76 (56.03%) Multiparity = 59 (43.97%)	0.18
Personal history of PE (n/%)	Yes = 6 (8.62%)	Yes = 0 (0%)	<0.001
Personal history of hypertension (n/%)	Yes = 6 (8.62%)	Yes = 11 (0.86%)	0.005
Personal history of renal disease (n/%)	Yes = 12 (1.72%)	Yes = 0 (0%)	0.15
Personal history of SLE/APS (n/%)	Yes = 5 (7.76%)	Yes = 3 (2.59%)	0.07
Macrosomic fetus (n/%)	Yes = 10 (14.7%)	Yes = 3 (2.2%)	<0.001
Obesity (n/%)	Yes = 18 (26.72%)	Yes = 5 (4.31%)	<0.001
Retained placenta (n/%)	Yes = 6 (4.08%)	Yes = 1 (0.74%)	0.001
Labour arrest (n/%)	Yes = 21 (30.8%)	Yes = 4 (2.96%)	<0.001
PAS (n/%)	Yes = 7 (10.2%)	Yes = 0 (0%)	<0.001
Lacerations (n/%)	Yes = 17 (25%)	Yes = 5 (4.31%)	<0.001
Instrumental delivery (n/%)	Yes = 4 (5.88%)	Yes = 1 (0.74%)	0.02
Fetal demise (n/%)	Yes = 2 (26.72%)	Yes = 0 (0%)	0.22

Legend: n—number of patients; SD—standard deviation; PPH—postpartum hemorrhage; PE—preeclampsia; SLE—systemic lupus erythematosus; APS—antiphospholipid syndrome; PAS—placenta accrete spectrum disorder.

**Table 2:** Predictive performance of models based on machine learning.

ML Model	Type of PPH	TPR (%)	FNR (%)	PPV (%)	FDR (%)	Accuracy (%)	AUC Value	Precision	Recall	F1 Score
DT	PPH	94.1	5.9	91.4	8.6	92.8	0.93	0.91	0.94	0.93
	Class I	92.9	7.1	75	25	94.1	0.95	0.93	0.75	0.86
	Class II	66.7	33.3	92.9	7.1	88.2	0.80	0.93	0.93	0.93
	Class III	75	25	91.7	8.3	82.4	0.80	0.85	0.92	0.88
	Class IV	82.1	17.9	44.4	55.6	79.4	0.70	0.67	0.44	0.53
NB	PPH	96.3	3.7	96.4	3.6	98.6	0.98	0.96	0.96	0.98
	Class I	96.4	3.6	80	20	91.2	0.88	0.67	0.80	0.73
	Class II	33.3	66.7	87.1	12.9	85.3	0.72	0.96	0.87	0.92
	Class III	25	75	79.3	20.7	73.5	0.68	0.88	0.79	0.84
	Class IV	89.3	10.7	50	50	82.4	0.67	0.50	0.50	0.50
SVM	PPH	70.6	29.4	77.8	22.2	85.5	0.98	0.71	0.78	0.88
	Class I	96.4	3.6	80	20	91.2	0.91	0.67	0.80	0.73
	Class II	33.3	66.7	86.7	13.3	82.4	0.76	0.93	0.87	0.90
	Class III	37.5	62.5	80.8	19.2	70.6	0.49	0.81	0.81	0.81
	Class IV	85.7	14.3	20	80	73.5	0.64	0.17	0.20	0.18
RF	PPH	94.1	5.9	91.4	8.6	92.8	0.94	0.91	0.94	0.93
	Class I	92.9	7.1	71.4	28.6	91.2	0.94	0.83	0.71	0.77
	Class II	66.7	33.3	92.9	7.1	88.2	0.84	0.93	0.93	0.93
	Class III	87.5	12.5	94.4	5.6	70.6	0.79	0.65	0.94	0.77
	Class IV	85.7	14.3	33.3	66.7	76.5	0.76	0.33	0.33	0.33

Legend: PPH—postpartum hemorrhage; DT—decision tree; NB—naïve Bayes; SVM—support vector machine; RF—random forest; TPR—true positive rate; FNR—false negative rate; PPV—positive predictive value; FDR—false detection rate; AUC—area under the receiver curve value.

## Data Availability

The data presented in this study are available on request from the corresponding author. The data are not publicly available due to local policies.
